# A Meadow of Glomerular Cysts in Chronic Obstructive Nephropathy

**DOI:** 10.1016/j.xkme.2026.101337

**Published:** 2026-03-14

**Authors:** Shoya Taniguchi, Yuki Oba, Katsuyuki Miki, Kei Kono, Yoshifumi Ubara, Kenichi Ohashi, Yuki Nakamura, Naoki Sawa

**Affiliations:** 1 Nephrology Center, Toranomon Hospital Kajigaya, Kanagawa, Japan; 2 Department of Pathology, Toranomon Hospital, Tokyo, Japan; 3 Department of Human Pathology, Institute of Science of Tokyo, Tokyo, Japan

**Figure 1:**
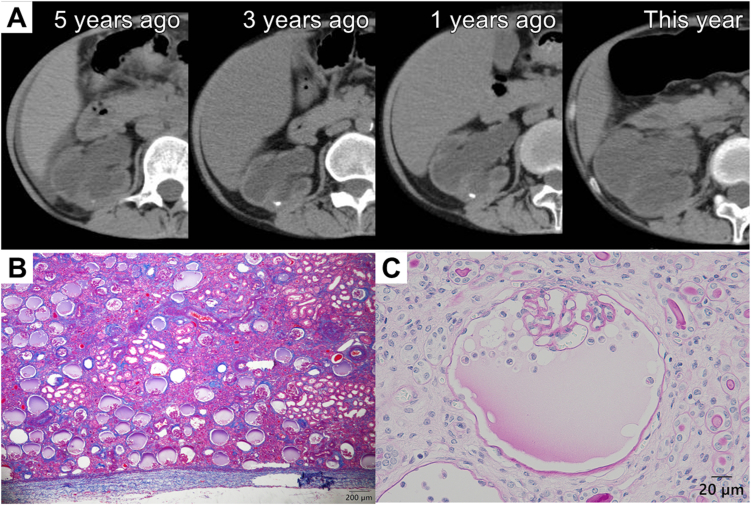
Description TK. (A) A plain abdominal computed tomography showed hydronephrosis in the right kidney, with surrounding fat stranding suggestive of pyelonephritis. Hydronephrosis is progressively worsening over time. (B) Light microscopy showed numerous glomerular cysts. Interstitial fibrosis was severe (Azan stain; original magnification, ×100). (C) Tamm-Horsfall protein, accompanied by podocyte, was observed refluxing into the Bowman's space (Periodic acid-Schiff stain; original magnification, ×400).

A 41-year-old Japanese woman with end-stage kidney disease due to type 1 renal tubular acidosis was performed an ABO-incompatible living-donor kidney transplantation from her mother 5 years ago. Although there had been no clear rejection and kidney function had been maintained up until this time, she admitted for prerenal acute kidney injury due to severe dehydration. She was treated with temporary hemodialysis and extracellular fluid administration. On hospital day nine, she developed a fever and right flank pain. The abdominal computed tomography showed the native right kidney had shown mild hydronephrosis before transplantation, but retrospective imaging revealed that hydronephrosis had progressively worsened over time and a large ureteral calculus was impacted in the right ureter (Fig 1A). She was diagnosed with calculous pyelonephritis on chronic hydronephrosis. The next day, percutaneous nephrostomy was performed, followed by an elective right native nephrectomy. Its light microscopic findings are shown in Fig 1B and C. The most striking finding in the excised specimen was the formation of numerous glomerular cysts. The space of Bowman's capsule was filled with refluxed Tamm–Horsfall protein and podocyte.

Iida et al^1^ reported a case of acute obstructive nephropathy in a renal allograft. However, their observations were based on a needle biopsy specimen, and the sampling window was therefore limited. Moreover, because the process was acute, reflux of Tamm–Horsfall protein into Bowman’s space was only scant. Many glomerular capillary tufts were preserved. This indicates that the renal injury was a hydrostatic injury caused by urinary tract obstruction, unlike glomerulonephritis accompanied by cell proliferation. As a unilateral ureteral obstruction model has demonstrated, the pathophysiology of obstructive nephropathy has been regarded as renal interstitial fibrosis.^2^ Our case shows chronic obstructive nephropathy that evolved over 5 years, captured in a nephrectomy specimen with such a diffuse distribution of glomerular cysts, and also demonstrates glomerular capillary tufts still persist within the glomerular cysts.
